# PERG adaptation for detection of retinal ganglion cell dysfunction in glaucoma: a pilot diagnostic accuracy study

**DOI:** 10.1038/s41598-021-02048-x

**Published:** 2021-11-24

**Authors:** T. Salgarello, G. M. Cozzupoli, A. Giudiceandrea, A. Fadda, G. Placidi, E. De Siena, F. Amore, S. Rizzo, B. Falsini

**Affiliations:** 1 Institute of Ophthalmology, Fondazione Policlinico Universitario A. Gemelli IRCCS - Università Cattolica del Sacro Cuore, Largo A. Gemelli 8, 00168 Rome, Italy; 2 Institute of Ophthalmology, Università Cattolica del Sacro Cuore, Rome, Italy; 3 Eye Center, Istituto Clinico Humanitas, Via Alessandro Manzoni, 56, 20089 Rozzano Milano, Italy; 4 Department of Cardiovascular, Dysmetabolic and Aging-Associated Diseases, Istituto Superiore di Sanità, Rome, Italy; 5 National Centre of Services and Research for the Prevention of Blindness and Rehabilitation of Low Vision Patients, Fondazione Policlinico Universitario A. Gemelli IRCCS, Rome, Italy

**Keywords:** Optic nerve diseases, Ocular hypertension, Glaucoma, Cell death in the nervous system

## Abstract

It has been previously demonstrated that the adaptive phase changes of steady-state pattern electroretinogram (SS-PERG), recorded during 4-min presentation of patterned stimuli, are reduced in glaucoma suspects and patients compared to normal subjects. Our study aims at testing the hypothesis that adaptive changes of SS-PERG, recorded using the novel optimized Next Generation PERG (PERGx) protocol, differ between glaucoma patients and controls. In this pilot cross-sectional study, we included 28 glaucoma patients and 17 age-matched normal subjects. Both patients and controls underwent a full ophthalmologic examination, visual field testing, OCT and PERGx. The PERGx signal was sampled over 2 min (providing 1 noise and 9 signal packets) in response to alternating gratings generated on an OLED display. PERGx amplitude and phase were analyzed to quantify adaptive changes over recording time. Receiver operating characteristic (ROC) curves were used to study the diagnostic accuracy of PERGx parameters in distinguishing glaucoma patients from normal subjects. PERGx amplitude and phase data showed declining trends in both groups. PERGx amplitude slope and grand-average vector amplitude and phase were significantly different in patients compared to controls (*p* < 0.01), whereas phase angular dispersion was greater in patients but not significantly different between the two groups. The area under the ROC curves were 0.87 and 0.76 for PERGx amplitude slope and grand-average vector amplitude, and 0.62 and 0.87 for PERGx angular dispersion and grand-average vector phase, respectively. The PERGx paradigm resulted highly accurate in detecting the reduction of amplitude adaptive changes in glaucoma patients, presumably due to the loss of functional retinal ganglion cell autoregulation. Thus, PERG adaptation, recorded by this new protocol, might be helpful in the identification and diagnosis of early glaucomatous dysfunction.

## Introduction

Retinal ganglion cell (RGC) death by apoptosis is the final pathway underlying all types of injuries to the optic nerve, including glaucoma. Several mechanisms are implied in the pathogenesis of glaucomatous damage and contribute to RGC loss, such as mechanical stretching of the lamina cribrosa, glial cell activation, oxidative stress, mitochondrial dysfunction, excitotoxicity, inflammation, immune response dysregulation, and protein misfolding^1,2^. However, it is largely accepted that there is an early stage of reversible RGC dysfunction preceding cell death^3,4^. Indeed, before RGC axons are completely damaged and RGCs have died there is a temporal window characterized by ultrastructural changes (synaptic dysfunction, dendritic pruning, axonal transport deficits and microglial proliferation) and reduced functional capacity (detectable as pattern electroretinogram [PERG] amplitude reduction)^5,6^. During this critical period RGCs are likely to adopt compensatory and successive rescue mechanisms in a stepwise process, until stress exposure exceeds their survival capacity^4^.

PERG is a noninvasive tool that has been shown to reflect RGC function in humans and experimental animals^7,8^. Several studies have demonstrated that PERG is already altered in early glaucoma patients^9–11^ and even often in glaucoma suspects^10–12^, so reflecting the dysfunctional state that precedes RGC death. In 2004, Porciatti et al.^13^ developed a novel paradigm for the steady-state PERG (SS-PERG) optimized for glaucoma screening, called PERGLA, and they found moderate correlations between its amplitude abnormalities and risk factors for primary open-angle glaucoma (POAG) in glaucoma suspects, suggesting that PERG may have a predictive potential for glaucoma development^10^.

Finally, a study^14^ conducted on 28 normal eyes demonstrated that the PERG response evoked by continuous stimulation with fast-reversing, high-contrast, patterned fields, shows a slow exponential decrease of its amplitude towards a plateau, reached after about 110 s. This adaptation effect, also known as “habituation”^15,16^, has been interpreted as the inner retina adjustment to changed metabolic requirements during a sustained stimulation. The response decline during habituation amounts to nearly 30% of the initial value. As a result, the PERG amplitude has a negative slope in normal eyes when plotted as a function of time^15^. Conversely, this adapting behavior was not detected by using the uniform flicker ERG^15^, maybe because of different retinal generators^8^. Altogether, these findings imply that prolonged steady-state pattern visual stimuli induce cell activity habituation effect reflecting a neurovascular/neurometabolic coupling at the RGCs level^17^.

In this regard, the clinical application of such stimulation has been also investigated in glaucoma^18^, by focusing on possible differences among early POAG patients, glaucoma suspects and normal controls. It has been shown that the magnitude of adaptive PERG phase changes significantly decreased with increasing severity of disease, whereas adaptive PERG amplitude changes were similar in the three groups of the study population. Recently, Monsalve et al.^19^ described a new upgraded protocol for adaptive SS-PERG change recording, dubbed Next Generation PERG (PERGx as a contraction of PERGnext), whose parameters strictly correlated with those of PERGLA, so allowing to be proposed as a promising source of further clinical information about RGC function in optic nerve diseases.

The aim of this study was to test the hypothesis that adaptive changes of the SS-PERG, recorded using the novel PERGx protocol, differ between glaucoma patients and normal subjects.

## Methods

A cross-sectional study was performed on consecutive patients presenting to the Glaucoma Service at the Fondazione Policlinico Universitario A. Gemelli IRCCS - Università Cattolica del Sacro Cuore of Roma, Italy, from April to June 2021. The study protocol (ID 3934) was approved by the Ethics Committee of the aforementioned institution and carried out in accordance with the tenets of the Declaration of Helsinki. Written informed consent was obtained from all subjects following an explanation of the nature and intent of the study.

### Subjects

The study population included a group of 28 glaucoma patients consisting of pre-perimetric, early and moderate stage patients (64.3% women and 35.7% men; mean age ± standard deviation [SD]: 58.64 ± 14.04 years, range 40–80). Seventeen normal subjects, whose sex and age distribution were comparable with those of patients, were also enrolled into the study as a control group.

Both patients and controls underwent a full ophthalmologic examination, including best-corrected Snellen visual acuity measurement, slit-lamp biomicroscopy of the ocular anterior segment and fundus, Goldmann applanation tonometry, and gonioscopy. Moreover, central corneal pachymetry by the digital ultrasonic pachymeter Pachmate DGH55 (DGH Technology, Inc., Exton PA, USA), computerized white-on-white 30–2 visual field testing by Humphrey Field Analyzer 750i (HFA; Carl Zeiss Meditec, Inc., Dublin CA, USA), SS-PERG with adaptation paradigm recording by Retimax (CSO, Florence, Italy), and measurement of both peripapillary retinal nerve fiber layer (RNFL) and macular ganglion cell/inner plexiform layer (GCIPL) thicknesses by spectral-domain Cirrus HD-OCT (model 5000, sw. version 10.0, Carl Zeiss Meditech, Inc., Dublin CA, USA) were also performed. Visual field, PERG, and OCT analyses were obtained for each subject within 1 week of each other.

Inclusion criteria were as follows: age between 40 and 80 years, normal range central corneal thickness values (520–570 μm), and fulfillment of all current clinical practice criteria used to diagnose POAG patients: (1) elevated intraocular pressure (IOP) at diagnosis (> 21 mmHg on two separate occasions); (2) open anterior chamber angle assessed by gonioscopy; (3) abnormal optic disc as defined on routine stereoscopic examination with slit-lamp biomicroscopy and 78-diopter (D) lens by vertical cup/disc (C/D) diameter ratio > 0.6 in medium sized discs (otherwise corrected for disc size, by considering the positive linear relationship between vertical C/D ratio and vertical disc diameter)^20^ and/or an interocular C/D diameter ratio asymmetry ≥ 0.2 unexplained by side differences in disc size, diffuse or focal rim thinning, notching; (4) reliable and reproducible visual field abnormalities (see below) accomplishing the Hodapp-Parrish-Anderson criteria^21^ for early to moderate glaucoma stages (based on the extent of glaucomatous damage expressed by mean deviation [MD] defect and the number and position of defective points), as well as normal visual field for pre-perimetric glaucoma. In pre-perimetric glaucoma eyes (60.71%) MD ± SD was 0.64 ± 1.14 dB, in early glaucoma eyes (32.14%) MD ± SD was − 1.58 ± 2.08 dB, and in moderate glaucoma eyes (7.14%) MD ± SD was − 10.95 ± 0.94 dB.

Two independent glaucoma experts (A.G. and T.S.) observed and defined the damage to the optic nerve head as detected by fundus examination. The inter-observer agreement coefficient was 91.7% (95% confidence interval = 83.7–99.7). In case of disagreement the specialists discussed together, sometimes by matching their clinical judgment with OCT analysis, mainly from RNFL thickness and deviation maps, until they eventually obtained a consensus. In our patient’s sample, as for Cirrus HD-OCT classification by the average RNFL thickness parameter, among pre-perimetric glaucoma patients, 68.75% of eyes were “outside normal limits” (thinner than all but 1% of normative database), 18.75% of eyes were “suspect” (thinner than all but 5% of normative database), and 12.50% of eyes were “within normal limits”, but in presence of a focal narrow defect at RNFL thickness and/or deviation map. Among early glaucoma patients, 88.89% of eyes were “outside normal limits” and 11.11% of eyes were “suspect”. Among moderate glaucoma patients, all eyes were “outside normal limits”.

All patients were under treatment with one or more topical hypotensive drugs (β-blockers, prostaglandin analogues, carbonic anhydrase inhibitors, and α_2_-agonists) providing a stable IOP lower than 21 mmHg, and sometimes neuroprotectants.

Exclusion criteria were as follows: corrected Snellen visual acuity < 20/25, refractive errors equal to or more than 2 D of myopia or hyperopia and 1 D of astigmatism, optic disc pallor exceeding cupping, cataract surgery or changes in the IOP-lowering and/or neuroprotectant therapies within the 3 months before patient recruitment and morpho-functional assessment, low perimetric reliability, and ophthalmologic or neurologic diseases which may affect visual function and exam execution.

The study can be considered as a diagnostic accuracy study according to STARD guidelines^22^, where the gold target condition was glaucoma diagnosis, the clinical reference standards were the clinical tests for glaucoma diagnosis (see below), and the index test was the PERG adaptation paradigm (PERGx). Data collection was planned before the reference standard tests and after the index tests were performed. Controls and patients participating in the study formed a random series. The investigators (B.F., G.P. and E.D.S.) performing the index test were masked as to the clinical diagnosis and the reference standard tests.

### Perimetry

Visual field sensitivity was determined for each eye using the HFA central 30-2 SITA-standard test. Only visual field exams with good reliability indices (fixation losses, false positive and negative errors < 20%)^23^ were evaluated. Abnormal perimetry was defined as a typical reproducible defect (arcuate and/or paracentral scotoma or nasal step) in three consecutive exams^24^, with one or more of the following alterations: Glaucoma Hemifield Test outside normal limits, pattern standard deviation (PSD) with *p* < 5%, and a cluster of ≥ 3 adjacent points, not contiguous with the field borders nor the blind spot, in the upper and/or lower hemifield of the total and pattern deviation plots with *p* < 5%, one of which reached *p* < 1%. For data analysis, the two global indices of field sensitivity, MD and PSD, were collected.

### OCT

OCT imaging was performed using the Cirrus HD-OCT on both peripapillary RNFL and macular GCIPL. The OCT lens was adjusted for the refractive error. The subject was instructed to stare at the internal fixation target with the eye under examination, to enable the optic disc and the macula to subsequently come into the appropriate windows and to be centered. The scan protocols were the Optic Disc Cube 200 × 200 and Macular Cube 512 × 128 for the study of peripapillary RNFL and macular GCIPL, respectively. After optimizing the reflective signal, three separate scans were obtained per eye by each protocol during the same session, and the best one with optimal signal strength (> 6/10) and scan image centering, no movements during scans or anomalous internal/external boundary definition was used for the analysis. Average RNFL and GCIPL thicknesses were collected.

### PERGx recording and analysis

The PERG was acquired simultaneously from both eyes with standard skin surface electrodes (Grass gold, 10 mm diameter) taped on the lower eyelids (active), ipsilateral temples (reference), and central forehead (ground) using the Retimax system. Subjects fixated at the center of the stimulating field (size, 60° width × 50° height) with natural pupils, whose size was measured (mean value, 3.5 ± 1.0 mm) at a viewing distance of 57 cm wearing full refractive correction. No statistically significant differences in pupil size were observed between patients and normal subjects. Fixation was monitored by a trained observer. Signals were amplified (gain of 100 k, 1–250 Hz bandwidth, 6 dB/octave slope), digitized (12 bit resolution, 2 kHz sampling rate, 100 µV AC range) and averaged in synchronism with stimulus onset. Artifacts, mainly from blinks or large eye movements, were automatically rejected to minimize amplitude bias.

PERGx was recorded similarly to a published protocol^19^. In particular, SS-PERG was elicited by black-and-white horizontal gratings of 0.8 cycles/degree spatial frequency and 95% contrast (mean luminance: 35 cd/m^2^), modulated in counterphase at 7.5 Hz (15 reversals/s). Stimulus was electronically generated on a high-resolution organic light-emitting diode television (OLED TV) monitor and administered continuously over nearly 2 min.

The response was recorded as a sequence of 10 partial averages (packets), each one (10 s average duration) obtained summing up to 60 cycles.^18^ The first packet was obtained with the patient exposed to a uniform gray stimulus equiluminant with the pattern stimulus, to obtain a “noise” response. Two replications of the entire adaptation paradigm, including noise level assessment, were recorded, and an appropriate time interval between replications was chosen to avoid residual adaptive effects.

A discrete Fourier analysis was performed on the recordings in order to isolate the PERG second harmonic (2P, the significant outcome of PERG experiments)^25^. The resulting waveforms (9 for each patient, excluding the first noise waveform) were further analyzed by plotting 2P amplitude and phase as a function of time for each subject. At the end of the procedure each response consisted of 9 second harmonic packets. Furthermore, PERGx amplitude and phase values were averaged across group subjects both for single packets (average scalar amplitude and phase) as well as for each response over the 9 packets (grand-average vector amplitude and phase). We assumed that the grand-average vector parameters represented an index of non-adapted RGC activity and corresponded to the ordinary SS-PERG^15^.

In addition to the grand-average measurements, we studied the PERGx habituation in terms of amplitude slope and phase angular dispersion. With regard to the former, a linear regression analysis was automatically applied to the PERGx vector amplitude plotted as a function of packets’ order number from each patient, to determine the slope (angular coefficient of linear function) of the adaptive amplitude changes. The residuals of this regression provided an estimate of the “noise”, that is the component of variance not attributable to the adaptive modifications. Angular dispersion^26^ was considered as an index of PERGx phase variability during the habituation process.

### Statistical analysis

Only the right eyes were considered. The following PERGx parameters were chosen as the primary outcome measures of the study: average scalar amplitude and phase for each packet; amplitude slope and phase angular dispersion, as measures of adaptive PERGx changes; and grand-average vector amplitude and phase, as surrogates of ordinary non-adapted SS-PERG. The secondary outcomes were the OCT morphometric parameters of peripapillary and macular retina, namely the RNFL and GCIPL thicknesses.

Statistical analysis was performed using SPSS 17.0 for Windows (IBM SPSS, Armonk NY, USA) and Origin 6.0 (Microcal Origin, Microcal Software Inc., Northampton MA, USA). Alpha and beta error were established at 5% and 20%, respectively. The following variables were considered as continuous quantitative variables: age; IOP measurement; perimetric MD and PSD indices; OCT RNFL and GCIPL thicknesses; PERGx amplitude slope, grand-average vector amplitude, phase angular dispersion and grand-average vector phase. Assimilability to normal distribution was evaluated using the Kolmogorov–Smirnov test.

The electrophysiological 9 signal packets were separately analyzed and PERGx amplitude and phase were initially studied as individual temporal series, and then averaged across subjects and plotted as a function of the single sequential packets. Linear regression analyses were applied to the amplitude and phase data in order to evaluate the presence of an adaptive behavior.

Univariate comparison between the two groups’ parameters was performed using the two-tailed Student’s *t*-test for independent groups. A Bonferroni corrected *p* value < 0.05 was considered to establish the statistical significance of the results. Receiver operating characteristic (ROC) curves were used to study the diagnostic accuracy of PERGx parameters (i.e. their ability to differentiate between unhealthy and healthy eyes) by evaluating the area under the curve (AUC), with an AUC of 0.5 indicating no discrimination ability and an AUC of 1.0 indicating maximal discrimination ability.

## Results

Eleven right early to moderate glaucoma eyes (39.29%) and 17 right pre-perimetric glaucoma eyes (60.71%) were considered.

Results from the descriptive analysis of the study groups are summarized in Table 1. No statistically significant differences between the 2 groups were detected in terms of age, gender, and IOP at the enrollment. With regard to the visual field test parameters, the patient group showed significantly (*p* < 0.05) different MD and PSD values compared to control group.

**Table 1:** Demographical and clinical characteristics of control group and glaucoma group.

	Control subjects (n = 17)	Glaucoma patients (n = 28)	*p*
Age, y: mean ± SD (*range*)	53.94 ± 5.75 *(44–64)*	58.64 ± 14.04 *(40–80)*	n.s.
Sex			
Male, No. (%)	6 (35.3)	18 (35.7)	n.s.
Female, No. (%)	11 (64.7)	10 (64.3)	n.s.
IOP, mmHg: mean ± SD (*range*)	14.88 ± 2.31 *(10–19)*	17.31 ± 3.98 *(11–24)*	n.s.
Visual field parameters, dB: mean ± SD (*range*)			
MD	0.94 ± 0.81 *(0.53–2.16)*	− 1.31 ± 3.79 *(−11.61 to 2.55)*	**< 0.05**
PSD	1.82 ± 0.67 *(1.37–2.36)*	2.91 ± 2.71 *(1.20–11.53)*	**< 0.05**

The IOP values reported for the patient group are intended as treated-IOP values. The statistical significance (*p*-value) of *t*-test comparisons between group parameters is shown.

In all subjects, the 2P signal component of PERGx was sufficiently above the noise level (signal/noise > 2.5, with the noise level at the 2P frequency ranging from 0.07 to 0.12 µV).

**Figure 1:**
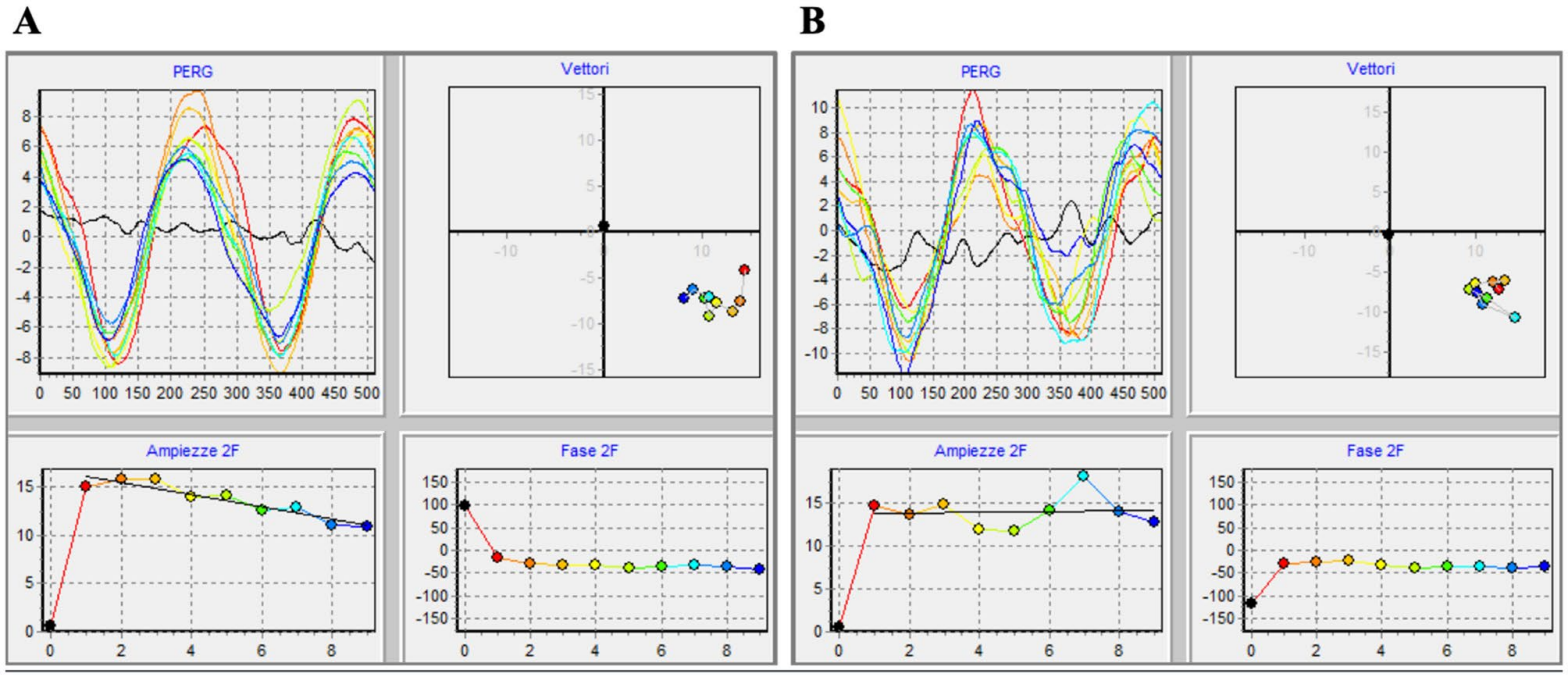
Representative examples of sequential PERGx samples recorded in two random subjects from control group (**A** top left panel) and patient group (**B** top left panel). The black waveform represents the noise recorded during an initial 10-s presentation of a grey uniform background, whereas the 9 coloured superimposed waveforms correspond to the responses obtained by high-contrast reversing black-and-white gratings. Data represent successive averages of 60 epochs each (~ 10 s sampling time). Top right graphs show PERGx vector changes over the recording time in the control subject (**A** top right panel) and the patient (**B** top right panel), respectively. The bottom graphs display how the PERGx amplitude and phase of successive samples (filled coloured symbols) change over time in the control (**A**) and in the patient (**B**), respectively.

Typical examples of PERG responses and their changes over time, recorded from a normal control subject and a glaucoma patient are shown in Fig. 1. In the normal control (Fig. 1A) PERGx amplitude declined considerably over time, whereas in the glaucoma patient (Fig. 1B) the decline was much less steep. PERGx phase changed little over continuous stimulation, but it was consistently more advanced in the glaucoma patient compared to the control.

**Figure 2:**
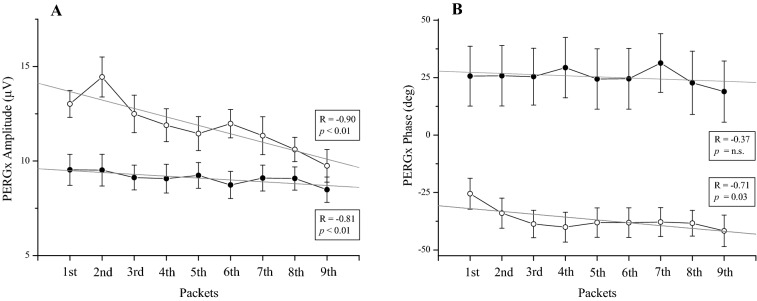
Scatter plots of scalar 2P amplitude (**A**) and phase (**B**) averaged across all subjects of both control (open circles) and patient (filled circles) groups as a function of packet number. The linear regression (R and *p* values are shown) applied to the amplitude and phase data shows a steeper decline (i.e. more negative slope) in controls compared with patients.

In Fig. 2, the PERGx plots show the time course of 2P scalar amplitude and phase in controls and patients from the 1st to 9th packet after excluding the preliminary noise data packet. In Fig. 2A, the typical decreasing trend of PERGx amplitude (slope = − 0.45, R = − 0.90, *p* = 0.001) was recognized in the control group. Patients showed a much smaller PERGx amplitude drop (slope = − 0.10, R = − 0.81, *p* = 0.008) over time compared to controls. In Fig. 2B, PERGx phase showed a negative slope over time in both controls (slope = − 1.24, R = − 0.71, *p* = 0.03) and patients (slope = − 0.49, R = − 0.37, *p* = n.s.), without statistical significance in the latter ones.

**Figure 3:**
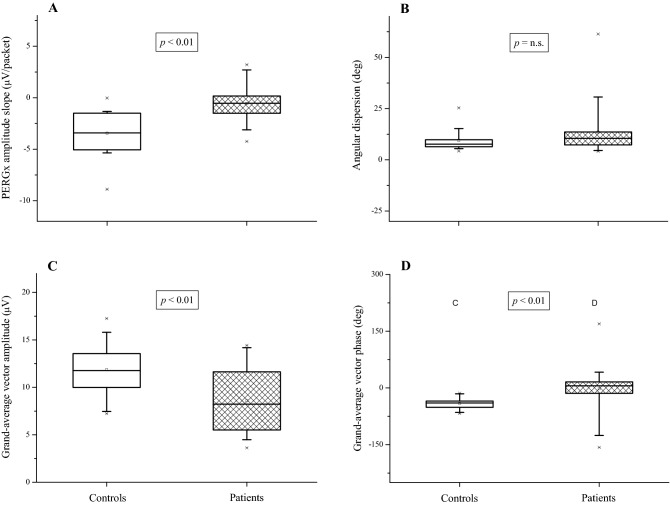
Box plots show the average, median and 25–75% percentiles of PERGx parameters, with whiskers and cross symbols representing the 5–95% and 1–99% percentiles, respectively. The statistical significance (*p*-value) of *t*-test comparisons between group parameters is shown.

Table 2 reports the primary and secondary outcomes in the two study groups as well as the statistical results of the *t*-test comparisons. Figure 3 displays separate box plots of the four primary outcome measures from each group. The slope of PERGx amplitude was significantly lower in absolute value (i.e. less negative) in patients compared to controls (*p* < 0.01) (Fig. 3A). The PERGx phase angular dispersion was greater in patients but not significantly different between the two groups (*p* = n.s.) (Fig. 3B). Both PERGx grand-average vector amplitude (Fig. 3C) and phase (Fig. 3D) were significantly different between the study groups (*p* < 0.01), with the former measure being lower and the latter one less negative in patients compared to controls.

**Table 2:** Descriptive analysis of the main outcome measures of the study.

Variable: mean ± SD (*range*)	Control subjects	Glaucoma patients	*p*
Amplitude slope (μV/packet)	− 3.44 ± 2.11 *(*−*8.89* to −*0.03)*	− 0.57 ± 1.66 *(*−*4.24* to *3.21)*	**< 0.01**
Phase angular dispersion (deg)	9.48 ± 5.08 *(4.28*−*25.34)*	13.52 ± 11.41 *(4.16*−*61.39)*	n.s.
Grand-average vector amplitude (μV)	11.88 ± 2.81 *(7.23* −*17.26)*	8.59 ± 3.34 *(3.62*−*14.42)*	**< 0.01**
Grand-average vector phase (deg)	− 41.05 ± 16.05 *(*−*67.33* to −*13.81)*	− 2.27 ± 55.10 *(*−*156.87* to *169.41)*	**< 0.01**
Average RNFL thickness (μm)	97.24 ± 6.63 *(85*−*113)*	91.43 ± 12.39 *(56*−*106)*	**< 0.05**
Average GCIPL thickness (μm)	80.76 ± 4.67 *(71* −*89)*	77.80 ± 8.31 *(58*−*90)*	n.s.

The statistical significance (*p* value) of *t*-test comparisons between group parameters is shown.

As for the secondary morphometric measurements, the patient group revealed lower average RNFL and GCIPL thicknesses compared to controls, reaching the statistical significance (*p* < 0.05) only in RNFL values.

**Figure 4:**
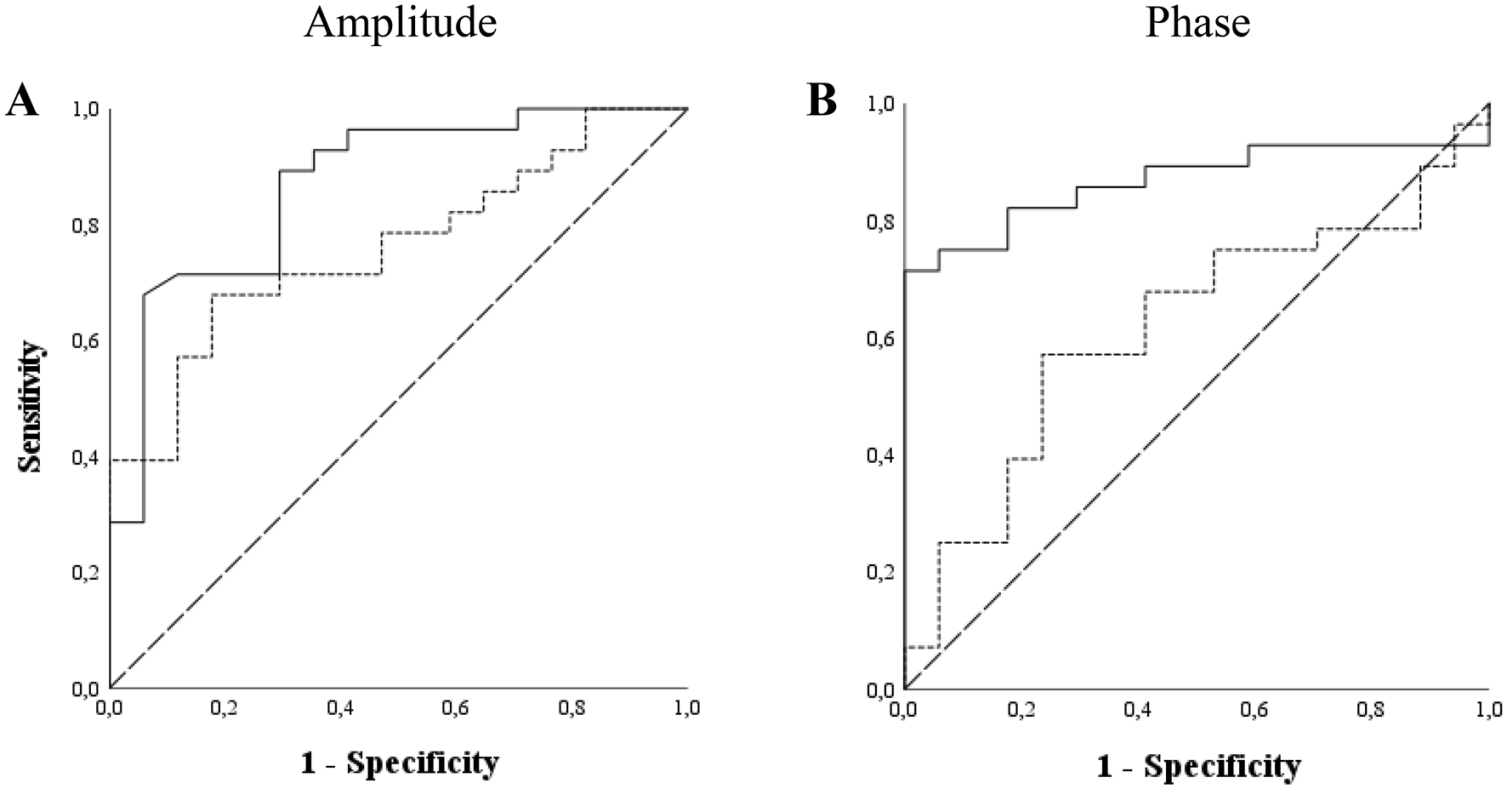
Receiver Operating Characteristic (ROC) curve and Area Under the Curve (AUC) calculated for (**A**) PERGx amplitude slope (solid line; AUC = 0.87) and grand-average vector amplitude (densely dashed line; AUC = 0.76) and for (**B**) PERGx grand-average vector phase (solid line; AUC = 0.87) and phase angular dispersion (densely dashed line; AUC = 0.62). The dashed diagonal line serves as an imaginary reference line representing a non-discriminatory test.

ROC curves were calculated for PERGx amplitude slope, grand-average vector amplitude and phase, and phase angular dispersion of patients and controls. The AUCs of PERGx amplitude parameters (Fig. 4A) were 0.87 (standard error [SE], 0.06; asymptotic *p* < 0.01; confidence limits, 0.76–0.98) for the amplitude slope and 0.76 (SE, 0.07; asymptotic *p* < 0.05; confidence limits, 0.62–0.90) for the grand-average vector amplitude. The AUCs of PERGx phase parameters (Fig. 4B) were 0.87 (SE, 0.06; asymptotic *p* < 0.01; confidence limits, 0.76–0.98) for the grand-average vector phase and 0.62 (SE, 0.09; asymptotic *p* = n.s.; confidence limits, 0.45–0.76) for the angular dispersion.

## Discussion

The present study was designed to compare the adaptive SS-PERG changes, as recorded by using an optimized paradigm called PERGx, between glaucoma patients and normal subjects. Furthermore, we wanted to determine if this tool was sufficiently accurate in discriminating patients from controls, and applicable to the clinical setting. Clearly, the study of PERG adaptation dynamics in already diagnosed glaucoma patients is more important for assessing the physiological status of RGCs, rather than for clinical diagnosis.

SS-PERG has been largely recognized as a reliable index of RGC function in patients with ocular hypertension or glaucoma^13,27,28^. In glaucoma, the SS-PERG at 16 reversals/s is consistently more reduced in amplitude than the transient PERG at 2 reversals/s^29^. This amplitude difference may be explained by the so-called energy budget model, according to which under steady state stimulus conditions (that is higher temporal frequencies) the metabolic demand of neurons may be greater than the available supply (energy budget).

Interestingly, if such energetically demanding conditions continue over time, a physiological adaptation phenomenon seems to occur in normal RGCs, as they reduce their activity to maintain a dynamic equilibrium compatible with their energy budget^13^. From an electrophysiological standpoint, when administering a prolonged pattern stimulus (for 100 s or more) adaptive changes take place in normal eyes, consisting in an exponential reduction of PERG amplitude, an effect often described by the term habituation^14^. The current protocol is specifically intended to explore the pathophysiology of RGC adaptation in glaucoma.

Adaptation has been recently interpreted as the autoregulatory ability of RGCs to reduce their activity following the exposure to visual stimuli causing in normal conditions high energy demand^30^. We may therefore consider our results in compromised glaucomatous retinas as evidence of a loss of adaptive capacity, which may not be efficient in meeting increased metabolic requirements. Previous studies have suggested that the impairment of habituation can be related to a loss of vascular autoregulation, mechanism well known in glaucoma^31,32^, and/or a lack of some neural mediators of autoregulation^33^. For this reason, habituation PERG represents an interesting source of additional biological information about RGC function in glaucoma patients, compared to the standard SS-PERG.

To the best of our knowledge, Porciatti et al.^18^ were the only ones to study the adaptation of SS-PERG in glaucoma. Notably, they demonstrated that adaptive PERG changes differed from standard SS-PERG responses (corresponding to the grand-average amplitude and phase measures) in glaucoma patients, maybe involving different functional dynamics and so providing further contribution to the study of impaired RGC activity. Moreover, they found that adaptive PERG phase changes significantly decreased with increasing severity of disease, whereas adaptive PERG amplitude changes were similar among glaucoma patients and control subjects. Conversely, as for the PERG grand-average values (amplitude and phase average across the sequential response packets from each subject), the amplitude decreased with increasing severity of disease, whereas the phase was not different among the three groups.

We used the fast PERGx protocol to assess the adaptive changes of the inner retina in the study population during the sustained presentation of a pattern stimulus, by recording two replications of the entire adaptation paradigm, including noise level assessment. The results showed a significantly reduced and less negative PERGx amplitude slope in patients compared to normal subjects, and a phase angular dispersion statistically not different between them. The PERGx grand-average vector amplitude was lower in patients compared to controls, contrary to the PERGx grand-average vector phase which was less negative, both reaching a statistical significance level. Notably, we observed a significant PERGx phase delay in patients compared to controls. The same change in response phase was found in previous studies by Porciatti and Ventura^10,12^. PERG phase delay in response latency may mean that active RGCs respond in a slower manner^12^.

As for adaptive PERGx changes, the present results agree with those reported by Porciatti et al.^18^, indicating a reduction of adaptive PERG changes in glaucoma, and hence a possible loss of functional RGC autoregulation. Nevertheless, we found a significantly lower amplitude slope in patients compared to controls as well as similar phase angular dispersion between the two groups. This discrepancy of our results compared to those from Porciatti et al. may be due to some differences in patient characteristics and recording protocol. For instance, Porciatti et al. conceived and used the first PERG habituation protocol, with longer presentation of the stimulus which created more stressful and energy consuming conditions for RGCs. The current protocol is an abbreviated and optimized tool designed to provide an easier clinical application along with adequate diagnostic accuracy.

Noteworthy, our study is the first to assess RGC dysfunction in glaucoma patients by using the PERGx paradigm proposed by Monsalve et al.^19^ ROC curve analyses demonstrated good diagnostic accuracy of the amplitude slope as adaptive PERGx parameter, reliably distinguishing glaucoma eyes from normal eyes. Thus, PERGx might be helpful in the detection and diagnosis of glaucomatous or pre-glaucomatous dysfunction. If the adaptive abnormalities are expression of a dysfunctional state and do not reflect a loss in the number of RGCs, then appropriate hypotensive or neuroprotective treatments might be able to rescue injured RGCs and restore adaptive PERG changes. Clinical pilot trials investigating the short-term effect of neuroprotection on adaptive PERG changes as an outcome variable will appropriately address this question.

## References

[1] Weinreb RN, Khaw PT (2004). Primary open-angle glaucoma. Lancet.

[2] Kolko M (2015). Present and new treatment strategies in the management of glaucoma. Open Ophthalmol. J..

[3] Porciatti V (2015). Electrophysiological assessment of retinal ganglion cell function. Exp. Eye Res..

[4] Porciatti V, Ventura LM (2012). Retinal ganglion cell functional plasticity and optic neuropathy: A comprehensive model. J. Neuro-Ophthalmol..

[5] Quigley HA, Dunkelberger GR, Green WR (1989). Retinal ganglion cell atrophy correlated with automated perimetry in human eyes with glaucoma. Am. J. Ophthalmol..

[6] Fry LE (2018). The coma in glaucoma: Retinal ganglion cell dysfunction and recovery. Prog. Retin. Eye Res..

[7] Porciatti V (2007). The mouse pattern electroretinogram. Doc. Ophthalmol..

[8] Zrenner E, Osborne NN, Chader CJ (1990). The physiological basis of the pattern electroretinogram. Progress in Retinal Research.

[9] Salgarello T (2018). Pattern electroretinogram detects localized glaucoma defects. Transl. Vis. Sci. Technol..

[10] Ventura LM, Porciatti V, Ishida K, Feuer WJ, Parrish RK (2005). Pattern electroretinogram abnormality and glaucoma. Ophthalmology.

[11] Ventura LM, Porciatti V (2006). Pattern electroretinogram in glaucoma. Curr. Opin. Ophthalmol..

[12] Ventura LM, Golubev I, Feuer WJ, Porciatti V (2013). Pattern electroretinogram progression in glaucoma suspects. J. Glaucoma.

[13] Porciatti V, Ventura LM (2004). Normative data for a user-friendly paradigm for pattern electroretinogram recording. Ophthalmology.

[14] Porciatti V, Sorokac N, Buchser W (2005). Habituation of retinal ganglion cell activity in response to steady state pattern visual stimuli in normal subjects. Invest. Ophthalmol. Vis. Sci..

[15] Fadda A (2009). Lack of habituation in the light adapted flicker electroretinogram of normal subjects : A comparison with pattern electroretinogram. Clin. Neurophysiol..

[16] Fadda A (2013). Reduced habituation of the retinal ganglion cell response to sustained pattern stimulation in multiple sclerosis patients. Clin. Neurophysiol..

[17] Riva CE, Logean E, Falsini B (2005). Visually evoked hemodynamical response and assessment of neurovascular coupling in the optic nerve and retina. Prog. Retin. Eye Res..

[18] Porciatti V (2014). Adaptation of the steady-state PERG in early glaucoma. J. Glaucoma.

[19] Monsalve P (2017). Next generation PERG method: Expanding the response dynamic range and capturing response adaptation. Transl. Vis. Sci. Technol..

[20] Hoffmann EM, Zangwill LM, Crowston JG, Weinreb RN (2007). Optic disk size and glaucoma. Surv. Ophthalmol..

[21] Hodapp E, Parrish RK, Anderson DR (1993). Clinical Decisions in Glaucoma.

[22] Bossuyt PM (2015). STARD 2015: an updated list of essential items for reporting diagnostic accuracy studies. Br. Med. J..

[23] Yohannan J (2017). Evidence-based criteria for assessment of visual field reliability. Ophthalmology.

[24] Keltner JL (2000). Confirmation of visual field abnormalities in the Ocular Hypertension Treatment Study. Arch. Ophthalmol..

[25] Holder GE (2007). ISCEV standard for clinical pattern electroretinography—2007 update. Doc. Ophthalmol..

[26] Zar J (1999). Biostatistical Anlysis.

[27] Falsini B, Marangoni D, Montrone L, Campagna F (2008). Structure–function relationship in ocular hypertension and glaucoma: Interindividual and interocular analysis by OCT and pattern ERG. Graefe’s Arch. Clin. Exp. Ophthalmol..

[28] Bach M (2006). Pattern ERG as an early glaucoma indicator in ocular hypertension: A long-term, prospective study. Invest. Ophthalmol. Vis. Sci..

[29] Bach M (2001). Electrophysiological approaches for early detection of glaucoma. Eur. J. Ophthalmol..

[30] Porciatti V, Chou TH (2021). Modeling retinal ganglion cell dysfunction in optic neuropathies. Cells.

[31] Moore D, Harris A, Wudunn D, Kheradiya N, Siesky B (2008). Dysfunctional regulation of ocular blood flow: A risk factor for glaucoma?. Clin. Ophthalmol..

[32] Chan KKW, Tang F, Tham CCY, Young AL, Cheung CY (2017). Retinal vasculature in glaucoma: a review. BMJ Open Ophthalmol..

[33] Pournaras CJ, Riva CE, Bresson-Dumont H, De Gottrau P, Bechetoille A (2004). Regulation of optic nerve head blood flow in normal tension glaucoma patients. Eur. J. Ophthalmol..

